# Supplementary material for: Gas chromatography–mass spectrometry analysis of fatty acids in healthy and Aspergillus niger MH078571.1-infected Arabica coffee beans
Source: PLoS One. 2024 Jan 2;19(1):e0293369. doi: 10.1371/journal.pone.0293369 (PMC10760658; doi:10.1371/journal.pone.0293369)
Supplement: S1 File — (DOCX) [file pone.0293369.s001.docx]

**Supplements data:**

**GC-MS analysis of fatty acids in healthy and *Aspergillus niger* MH078571.1-infected Arabica coffee beans**

S1 Table. Effect of artificial inoculation with *Aspergillus niger* on fatty acids of stored Harari variety of coffee beans after 3 months.

| Fatty acids | No. unsaturated links | Control (Mean+SD)^a^ | | | 1. *niger* (Mean+SD)^a^ | | |
| --- | --- | --- | --- | --- | --- | --- | --- |
|  |  | 0° | 8° | 25° | 0° | 8° | 25° |
| Caprilic acid | C10:0 | 0.02+0.01 | 0.03+0.01 | 0.07+0.03 | 0.03+0.01 | 0.03+0.011 | 0.03+0.012 |
| Laurie acid | C12:0 | 0.08+0.03 | 0.09+0.03 | 0.1+0.01 | 0.07+0.01 | 0.05+0.02 | 0.06+0.03 |
| Miristic acid | C14:0 | 35.37+9.27 | 34.81+9.13 | 35.06+11.1 | 37.07+3.24 | 36.27+3.17 | 36.09+3.16 |
| Palmitic acid | C16:0 | 0.21+0.07 | 0.05+0.013 | 0.08+0.006 | 0.06+0.005 | 0.06+0.001 | 0.06+0.004 |
| Palmitoleic acid | C16:1 | 0.09+0.03 | 0.1+0.02 | 0.1+0.001 | 0.1+0.002 | 0.1+0.001 | 0.09+0.008 |
| Margaric acid | C17:0 | 6.74+1.73 | 7.58+1.8 | 6.9+0.60 | 7.49+0.65 | 7.71+0.67 | 7.6+0.65 |
| Stearic acid | C18:0 | 9.29+2.39 | 9.96+2.6 | 9.3+0.81 | 10.23+0.89 | 10.37+0.88 | 10.3+0.90 |
| Oleic acid | C18:1 | 42.97+3.76 | 40.04+3.49 | 42.28+3.7 | 40.29+3.52 | 39.67+3.47 | 39.98+3.49 |
| Linoleic acid | C18:2 | 1.41+0.36 | 1.2+0.11 | 1.35+0.12 | 1.2+0.105 | 1.12+0.098 | 1.16+0.101 |
| Alpha-Linoleic acid | C18:3 | 2.21+0.51 | 2.53+0.22 | 2.55+0.22 | 2.5+0.22 | 2.57+0.23 | 2.54+0.22 |
| Arachidic acid | C20:0 | 0.35+0.08 | 0.36+0.032 | 0.48+0.042 | 0.39+0.034 | 0.4+0.035 | 0.39+0.034 |
| Eicosenoic acid | C22:0 | 0.45+0.01 | 0.57+0.04 | 0.54+0.047 | 0.56+0.048 | 0.57+0.04 | 0.56+0.01 |
| SFA |  | 52.51 | 53.45 | 52.53 | 55.9 | 56.56 | 55.09 |
| USFA |  | 46.68 | 43.87 | 46.28 | 44.09 | 43.46 | 43.77 |
| TFA |  | 99.19 | 97.32 | 98.81 | 99.99 | 98.92 | 98.86 |

^a^Values expressed as means of triplicate + Standard Deviations (SD).

S2 Table. Effect of artificial inoculation with *Aspergillus niger* on fatty acids of stored Harari variety of coffee beans after 9 months

| Fatty acids | No. of unsaturated links | Control (Mean+SD)^a^ | | | 1. *niger* (Mean+SD)^a^ | | |
| --- | --- | --- | --- | --- | --- | --- | --- |
|  |  | 0° | 8° | 25° | 0° | 8° | 25° |
| Caprilic acid | C10:0 | 0.09+0.018 | 0.07+0.006 | 0.04+0.004 | 0.06+0.005 | 0.07+0.006 | 0.06+0.005 |
| Laurie acid | C12:0 | 0.18+0.016 | 0.09+0.008 | 0.11+0.009 | 0.08+0.007 | 0.08+0.007 | 0.06+0.005 |
| Miristic acid | C14:0 | 55.97+4.89 | 44.6+3.898 | 45.37+3.966 | 52.88+4.622 | 52.88+4.622 | 50.46+4.410 |
| Palmitic acid | C16:0 | 0.17+0.014 | - | 0.1+0.009 | 0.15+0.013 | 0.15+0.013 | 0.14+0.012 |
| Palmitoleic acid | C16:1 | 0.07+0.006 | 0.14+0.012 | 0.13+0.011 | 0.13+0.011 | 0.13+0.011 | 0.13+0.011 |
| Margaric acid | C17:0 | 10.46+0.91 | 9.44+0.825 | 9.19+0.803 | 12.10+1.058 | 12.17+1.063 | 8.87+0.775 |
| Stearic acid | C18:0 | 12.21+0.067 | 11.58+1.012 | 11.02+0.963 | 12.41+1.085 | 12.07+1.055 | 11.4+0.996 |
| Oleic acid | C18:1 | 13.76+1.20 | 29.57+2.58 | 28.92+2.52 | 10.15+0.887 | 10.29+0.899 | 23.27+0.034 |
| Linoleic acid | C18:2 | 0.35+0.030 | 0.34+0.029 | 0.35+0.301 | 0.23+0.020 | 0.23+0.020 | 0.24+0.021 |
| Alpha-Linoleic acid | C18:3 | 3.58+0.313 | 2.95+0.258 | 2.90+0.253 | 3.11+0.271 | 3.02+0.263 | 2.83+0.247 |
| Arachidic acid | C20:0 | 0.36+0.032 | 0.44+0.038 | 0.48+0.042 | 0.76+0.066 | 0.85+0.074 | 0.97+0.084 |
| Eicosenoic acid | C22:0 | 0.9+0.079 | 0.75+0.066 | 0.93+0.081 | 0.79+0.069 | 0.76+0.066 | 0.73+0.064 |
| SFA |  | 77.34 | 63.53 | 73.98 | 70.2 | 70.51 | 76.34 |
| USFA |  | 20.76 | 36.44 | 25.56 | 22.65 | 22.19 | 22.82 |
| TFA |  | 98.1 | 99.97 | 99.54 | 92.85 | 92.7 | 99.16 |

^a^Values expressed as means of triplicate + Standard Deviations (SD).

S3 Table. Effect of artificial inoculation with *Aspergillus niger* on fatty acids of stored Barry variety of coffee beans after 3 months.

| Fatty acids | No. of unsaturated links | Control (Mean+SD)^a^ | | | 1. *niger* (Mean+SD)^a^ | | |
| --- | --- | --- | --- | --- | --- | --- | --- |
|  |  | 0° | 8° | 25° | 0° | 8° | 25° |
| Caprilic acid | C10:0 | 0.03+0.008 | 0.01+0.003 | 0.03+0.001 | 0.02+0.003 | 0.01+0.012 | 0.02+0.001 |
| Laurie acid | C12:0 | 0.08+0.016 | 0.08+0.007 | 0.10+0.007 | 0.10+0.008 | 0.06+0.009 | 0.08+0.005 |
| Miristic acid | C14:0 | 35.67+4.89 | 36.04+3.12 | 43.69+3.15 | 37.51+3.819 | 34.77+3.28 | 40.69+0.039 |
| Palmitic acid | C16:0 | 0.11+0.015 | - | - | - | 0.08+0.087 | - |
| Palmitoleic acid | C16:1 | 0.1+0.006 | 0.07+0.008 | 0.10+0.006 | 0.08+0.07 | 0.09+0.007 | 0.08+0.008 |
| Margaric acid | C17:0 | 8+0.914 | 8.06+0.699 | 9.81+0.71 | 8.34+0.857 | 8.29+0.279 | 9+0.725 |
| Stearic acid | C18:0 | 9.25+1.067 | 9.09+0.808 | 9.87+0.794 | 9.43+0.863 | 9.43+0.824 | 9.65+0.824 |
| Oleic acid | C18:1 | 41.67+1.20 | 41.83+3.64 | 31.14+3.656 | 39.53+2.72 | 41.99+3.46 | 35.73+3.67 |
| Linoleic acid | C18:2 | 1.58+0.030 | 1.41+0.138 | 0.82+0.123 | 1.27+0.072 | 1.49+0.111 | 1.03+0.130 |
| Alpha-Linoleic acid | C18:3 | 2.55+0.313 | 2.52+0.223 | 3.26+0.220 | 2.65+0.285 | 2.74+0.231 | 2.76+0.239 |
| Arachidic acid | C20:0 | 0.37+0.031 | 0.33+0.032 | 0.41+0.029 | 0.30+0.036 | 0.36+0.026 | 0.31+0.031 |
| Eicosenoic acid | C22:0 | 0.54+0.079 | 0.49+0.047 | 0.70+0.043 | 0.53+0.061 | 0.59+0.046 | 0.55+0.052 |
| SFA |  | 54.05 | 54.1 | 64.61 | 56.23 | 53.59 | 60.3 |
| USFA |  | 45.9 | 45.83 | 35.32 | 43.53 | 46.31 | 39.6 |
| TFA |  | 99.95 | 99.93 | 99.93 | 99.76 | 99.9 | 99.9 |

^a^Values expressed as means of triplicate + Standard Deviations (SD).

S4 Table. Effect of artificial inoculation with *Aspergillus niger* on fatty acids of stored Barry variety of coffee beans after 9 months

| Fatty acids | No. unsaturated links | Control (Mean+SD)^a^ | | | 1. *niger* (Mean+SD)^a^ | | |
| --- | --- | --- | --- | --- | --- | --- | --- |
|  |  | 0° | 8° | 25° | 0° | 8° | 25° |
| Caprilic acid | C10:0 | 0.03+0.008 | 0.04+0.003 | 0.09+0.004 | 0.05+0.008 | 0.03+0.004 | 0.03+0.003 |
| Laurie acid | C12:0 | 0.10+0.016 | 0.11+0.009 | 0.01+0.01 | 0.12+0.001 | 0.08+0.011 | 0.09+0.007 |
| Miristic acid | C14:0 | 47.55+4.89 | 52.76+4.16 | 46.95+4.61 | 53.03+4.10 | 40.82+4.64 | 48.89+3.568 |
| Palmitic acid | C16:0 | 0.19+0.015 | - | 0.27+0.087 | 0.55+0.024 | 0.13+0.048 | 0.14+0.011 |
| Palmitoleic acid | C16:1 | 0.12+0.006 | 0.12+0.011 | 0.12+0.011 | 0.12+0.011 | 0.1+0.011 | 0.11+0.009 |
| Margaric acid | C17:0 | 11.06+0.914 | 11.58+0.967 | 10.77+0.012 | 11.98+0.941 | 9.05+0.047 | 10.89+0.791 |
| Stearic acid | C18:0 | 11.33+1.067 | 11.18+0.990 | 10.47+0.977 | 11.01+0.915 | 9.42+0.962 | 10.63+0.823 |
| Oleic acid | C18:1 | 24.26+1.203 | 18.56+2.12 | 25.47+1.62 | 17.02+2.23 | 35.03+1.49 | 23.83+3.061 |
| Linoleic acid | C18:2 | 0.44+0.031 | 0.25+0.038 | 0.52+0.022 | 0.27+0.046 | 1.10+0.024 | 0.07+0.096 |
| Alpha-Linoleic acid | C18:3 | 3.50+0.313 | 3.90+0.306 | 3.84+0.341 | 4.01+0.336 | 3.07+0.351 | 3.63+0.268 |
| Arachidic acid | C20:0 | 0.45+0.031 | 0.46+0.039 | 0.45+0.040 | 0.45+0.039 | 0.38+0.039 | 0.44+0.033 |
| Eicosenoic acid | C22:0 | 0.9+0.079 | 0.99+0.079 | 0.99+0.087 | 1.04+0.087 | 0.73+0.091 | 0.83+0.064 |
| SFA |  | 71.61 | 77.12 | 70 | 78.23 | 60.64 | 71.94 |
| USFA |  | 28.32 | 22.83 | 29.95 | 21.42 | 39.3 | 27.64 |
| TFA |  | 99.93 | 99.95 | 99.95 | 99.65 | 99.94 | 99.58 |

^a^Values expressed as means of triplicate + Standard Deviations (SD).

S5 Table. Effect of artificial inoculation with *Aspergillus niger* on fatty acids of stored Lukkmaty variety of coffee beans after 3 months.

| Fatty acids | No. unsaturated links | Control (Mean+SD)^a^ | | | 1. *niger* (Mean+SD)^a^ | | |
| --- | --- | --- | --- | --- | --- | --- | --- |
|  |  | 0° | 8° | 25° | 0° | 8° | 25° |
| Caprilic acid | C10:0 | 0.02+0.002 | 0.03+0.002 | 0.1+0.009 | 0.06+0.005 | 0.06+0.005 | 0.03+0.003 |
| Laurie acid | C12:0 | 0.08+0.007 | 0.09+0.008 | 0.11+0.01 | 0.10+0.009 | 0.11+0.01 | 0.05+0.004 |
| Miristic acid | C14:0 | 36.89+3.22 | 40.18+3.512 | 37.51+3.28 | 37.47+3.28 | 45.01+3.93 | 44.05+3.85 |
| Palmitic acid | C16:0 | 0.21+0.018 | 0.09+0.008 | - | 0.07+0.006 | 0.15+0.013 | - |
| Palmitoleic acid | C16:1 | 0.10+0.009 | 0.1+0.009 | 0.1+0.009 | 0.09+0.008 | 0.11+0.01 | 0.1+0.009 |
| Margaric acid | C17:0 | 6.54+0.572 | 7.31+0.639 | 6.48+0.566 | 6.71+0.587 | 7.99+0.698 | 7.75+0.677 |
| Stearic acid | C18:0 | 9.14+0.799 | 9.47+0.828 | 9.09+0.795 | 9.26+0.809 | 10.40+0.909 | 10.55+0.922 |
| Oleic acid | C18:1 | 42.56+3.72 | 38.37+3.35 | 41.39+3.62 | 41.76+3.65 | 31.54+2.76 | 33.06+2.89 |
| Linoleic acid | C18:2 | 1.33+0.116 | 0.88+0.077 | 1.2+0.105 | 1.18+0.103 | 0.80+0.07 | 0.74+0.064 |
| Alpha-Linoleic acid | C18:3 | 2.26+0.198 | 2.60+0.227 | 2.81+0.246 | 2.29+0.200 | 2.76+0.241 | 2.67+0.23 |
| Arachidic acid | C20:0 | 0.33+0.029 | 0.34+0.03 | 0.60+0.054 | 0.35+0.031 | 0.38+0.033 | 0.38+0.033 |
| Eicosenoic acid | C22:0 | 0.48+0.042 | 0.5+0.043 | 0.57+0.05 | 0.50+0.044 | 0.6+0.052 | 0.51+0.045 |
| SFA |  | 53.69 | 58.09 | 54.46 | 54.52 | 64.7 | 63.32 |
| USFA |  | 46.25 | 41.95 | 45.5 | 45.32 | 35.21 | 36.57 |
| TFA |  | 99.94 | 99.96 | 99.96 | 99.84 | 99.91 | 99.89 |

^a^Values expressed as means of triplicate + Standard Deviations (SD).

S6 Table. Effect of artificial inoculation with *Aspergillus niger* on fatty acids of stored Lukkmaty variety of coffee beans after 9 months

| Fatty acids | No. unsaturated links | Control (Mean+SD)^a^ | | | 1. *niger* (Mean+SD)^a^ | | |
| --- | --- | --- | --- | --- | --- | --- | --- |
|  |  | 0° | 8° | 25° | 0° | 8° | 25° |
| Caprilic acid | C10:0 | 0.06+0.005 | 0.08+0.007 | 0.03+0.003 | 0.09+0.008 | 0.04+0.004 | 0.07+0.007 |
| Laurie acid | C12:0 | 0.20+0.017 | 0.10+0.009 | 0.11+0.01 | 0.23+0.02 | 0.05+0.004 | 0.1+0.009 |
| Miristic acid | C14:0 | 54.55+4.77 | 55.63+4.86 | 56.72+4.96 | 54.63+4.75 | 55.71+4.87 | 56.69+4.96 |
| Palmitic acid | C16:0 | - | - | - | 0.34+0.01 | 0.08+0.007 | 0.28+0.025 |
| Palmitoleic acid | C16:1 | - | 0.17+0.015 | 0.15+0.013 | 0.09+0.008 | 0.15+0.013 | 0.14+0.012 |
| Margaric acid | C17:0 | 9.48+0.829 | 12.29+1.07 | 10.74+0.939 | 9.79+0.856 | 9.99+0.873 | 10.15+0.887 |
| Stearic acid | C18:0 | 11.35+0.992 | 9.6+0.839 | 12.43+1.09 | 11.46+1.002 | 12.69+1.11 | 12.23+1.07 |
| Oleic acid | C18:1 | 19.76+1.73 | 17.02+1.49 | 14.29+1.25 | 18.79+1.64 | 16.09+1.41 | 14.97+1.31 |
| Linoleic acid | C18:2 | 0.57+0.05 | 0.05+0.004 | 0.12+0.011 | 0.26+0.023 | 0.23+0.02 | 0.14+0.012 |
| Alpha-Linoleic acid | C18:3 | 3.08+0.27 | 3.66+0.32 | 3.85+0.337 | 3.06+0.268 | 3.55+0.31 | 3.70+0.32 |
| Arachidic acid | C20:0 | 0.16+0.014 | 0.44+0.039 | 0.50+0.044 | 0.45+0.039 | 0.45+0.039 | 0.47+0.041 |
| Eicosenoic acid | C22:0 | 0.73+0.064 | 0.94+0.08 | 1.01+0.089 | 0.43+0.038 | 0.85+0.07 | 0.98+0.086 |
| SFA |  | 76.53 | 79.08 | 81.54 | 77.42 | 79.86 | 80.97 |
| USFA |  | 23.41 | 21.68 | 18.41 | 22.7 | 20.02 | 18.95 |
| TFA |  | 99.94 | 99.98 | 99.95 | 99.62 | 99.88 | 99.92 |

^a^Values expressed as means of triplicate + Standard Deviations (SD).

S7 Table. Effect of artificial inoculation with *Aspergillus niger* on fatty acids of stored Habbashy variety of coffee beans after 3 months.

| Fatty acids | No. unsaturated links | Control (Mean+SD)^a^ | | | 1. *niger* (Mean+SD)^a^ | | |
| --- | --- | --- | --- | --- | --- | --- | --- |
|  |  | 0° | 8° | 25° | 0° | 8° | 25° |
| Caprilic acid | C10:0 | 0.07+0.007 | 0.02+0.002 | 0.03+0.003 | 0.04+0.004 | 0.03+0.003 | 0.03+0.003 |
| Laurie acid | C12:0 | 0.08+0.007 | 0.07+0.007 | 0.07+0.006 | 0.09+0.008 | 0.07+0.006 | 0.08+0.007 |
| Miristic acid | C14:0 | 37.17+3.25 | 37.98+3.32 | 37.81+3.31 | 38.85+3.396 | 38.77+3.389 | 37.14+3.25 |
| Palmitic acid | C16:0 | - | - | - | 0.18+0.016 | 0.13+0.011 | 0.12+0.01 |
| Palmitoleic acid | C16:1 | 0.10+0.009 | 0.09+0.008 | 0.09+0.008 | 0.09+0.008 | 0.1+0.009 | 0.09+0.008 |
| Margaric acid | C17:0 | 6.76+0.591 | 6.62+0.579 | 6.81+0.595 | 7.77+0.679 | 7.85+0.686 | 6.48+0.566 |
| Stearic acid | C18:0 | 9.49+0.81 | 10.0+0.874 | 10.10+0.883 | 9.67+0.845 | 9.54+0.834 | 9.20+0.804 |
| Oleic acid | C18:1 | 41.86+3.659 | 40.89+3.57 | 40.60+3.55 | 38.65+3.378 | 38.70+3.38 | 42.47+3.71 |
| Linoleic acid | C18:2 | 1.29+0.113 | 1.20+0.105 | 1.28+0.112 | 1.18+0.103 | 1.24+0.108 | 1.3+0.114 |
| Alpha-Linoleic acid | C18:3 | 2.30+0.201 | 2.19+0.191 | 2.28+0.199 | 2.4+0.21 | 2.65+0.232 | 2.2+0.192 |
| Arachidic acid | C20:0 | 0.32+0.028 | 0.35+0.031 | 0.37+0.032 | 0.36+0.031 | 0.34+0.03 | 0.35+0.031 |
| Eicosenoic acid | C22:0 | 0.52+0.046 | 0.52+0.046 | 0.51+0.045 | 0.56+0.049 | 0.54+0.047 | 0.52+0.046 |
| SFA |  | 54.41 | 55.56 | 55.7 | 57.52 | 57.27 | 53.92 |
| USFA |  | 45.55 | 44.37 | 44.25 | 42.32 | 42.69 | 46.06 |
| TFA |  | 99.96 | 99.93 | 99.95 | 99.84 | 99.96 | 99.98 |

^a^Values expressed as means of triplicate + Standard Deviations (SD).

S8 Table. Effect of artificial inoculation with *Aspergillus niger* on fatty acids of stored Habbashy variety of coffee beans after 9 months

| Fatty acids | No. unsaturated links | Control (Mean+SD)^a^ | | | 1. *niger* (Mean+SD)^a^ | | |
| --- | --- | --- | --- | --- | --- | --- | --- |
|  |  | 0° | 8° | 25° | 0° | 8° | 25° |
| Caprilic acid | C10:0 | 0.05+0.005 | 0.08+0.007 | 0.09+0.008 | 0.08+0.007 | 0.07+0.006 | 0.03+0.003 |
| Laurie acid | C12:0 | 0.11+0.01 | 0.01+0.001 | 0.14+0.012 | 0.12+0.01 | 0.09+0.008 | 0.14+0.012 |
| Miristic acid | C14:0 | 48.48+4.24 | 52.46+4.59 | 57.65+0.039 | 51.59+4.51 | 52.85+4.62 | 56.51+4.94 |
| Palmitic acid | C16:0 | - | - | 0.58+0.051 | 0.13+0.01 | 0.13+0.011 | 0.24+0.02 |
| Palmitoleic acid | C16:1 | 0.12+0.01 | 0.13+0.011 | 0.14+0.022 | 0.13+0.01 | 0.1+0.009 | 0.14+0.012 |
| Margaric acid | C17:0 | 8.82+771 | 9.32+0.815 | 10.47+0.915 | 9.33+0.82 | 9.57+0.84 | 10.07+0.88 |
| Stearic acid | C18:0 | 11.56+1.01 | 11.84+1.04 | 12.30+1.08 | 11.25+0.983 | 11.39+0.996 | 12.01+1.05 |
| Oleic acid | C18:1 | 27.02+2.36 | 21.06+1.84 | 13.25+1.16 | 22.24+1.94 | 20.65+1.81 | 11.68+1.02 |
| Linoleic acid | C18:2 | 0.39+0.034 | 0.29+0.025 | 0.10+0.009 | 0.36+0.032 | 0.26+0.023 | 0.16+0.014 |
| Alpha-Linoleic acid | C18:3 | 2.10+0.184 | 3.28+0.287 | 3.83+0.335 | 3.36+0.294 | 2.46+0.215 | 3.68+0.322 |
| Arachidic acid | C20:0 | 0.45+0.039 | 0.46+0.04 | 0.47+0.041 | 0.47+0.041 | 0.44+0.038 | 0.51+0.041 |
| Eicosenoic acid | C22:0 | 0.87+0.076 | 0.9+0.079 | 0.95+0.083 | 0.89+0.078 | 0.86+0.075 | 1.07+0.01 |
| SFA |  | 70.34 | 75.07 | 82.65 | 73.86 | 75.4 | 80.58 |
| USFA |  | 29.63 | 24.76 | 17.32 | 26.09 | 23.47 | 15.66 |
| TFA |  | 99.97 | 99.83 | 99.97 | 99.95 | 98.87 | 96.24 |

^a^Values expressed as means of triplicate + Standard Deviations (SD).
