# Supplementary material for: Gas chromatography–mass spectrometry analysis of fatty acids in healthy and Aspergillus niger MH078571.1-infected Arabica coffee beans
Source: PLoS One. 2024 Jan 2;19(1):e0293369. doi: 10.1371/journal.pone.0293369 (PMC10760658; doi:10.1371/journal.pone.0293369)
Supplement: Attachment — Submitted filename: PONE-D-23-23915_reviewer.pdf [file pone.0293369.s002.pdf]

PLOS ONE

GC-MS analysis of fatty acids in healthy and Aspergillus niger MH078571.1-infected Arabica coffee beans.

--Manuscript Draft--

**Manuscript Number:**	PONE-D-23-23915
**Article Type:**	Research Article
**Full Title:**	GC-MS analysis of fatty acids in healthy and Aspergillus niger MH078571.1-infected Arabica coffee beans.
**Short Title:**	GC-MS analysis of fatty acids in infected Arabica coffee beans
**Corresponding Author:**	Amira H. Alabdalall, Associate of Professor<br>Imam Abdulrahman Bin Faisal University<br>Dammam, SAUDI ARABIA
**Keywords:**	Coffee beans; fatty acid; margaric; stearic; oleic; miristic; Aspergillus niger
**Abstract:**	The organic composition of Arabica coffee beans, particularly fatty acids, significantly influences their overall quality. After measuring its composition of fatty acids, it contained a high percentage of saturated fatty acids, including caprylic, lauric, myristic, palmitic, margaric, fat, and orchid. Moreover, the sample contained unsaturated fatty acids, namely palmitoleic acid (C16:1), oleic acid (C18:1), linoleic acid (C18:2), and alpha-linoleic acid (C18:3).<br>Coffee beans are susceptible to infection by fungi during storage, the development of which has adverse effects on the beans. The present study aimed to examine the impact of Aspergillus niger MH078571.1 infection on the diversity and abundance of fatty acids in green Arabica coffee beans. The impact of Aspergillus niger on the consumption of fatty acids in Arabica coffee beans was assessed. The findings of the study indicate that the duration of storage had a significant impact on the levels of fatty acids, specifically miristic (C14:0), margaric (C17:0), and stearic (C18:0), which increased as the storage period and temperature increased. Conversely, the percentage of oleic acid decreased under these conditions. This trend was observed across different storage temperatures (0, 8, and 25 °C) in untreated coffee beans affected by fungal activity.
**Order of Authors:**	Amira Hassan Alabdalall, Associate of Professor
**Additional Information:**	
**Question**	**Response**
**Financial Disclosure**<br><br>Enter a financial disclosure statement that describes the sources of funding for the work included in this submission. Review the submission guidelines for detailed requirements. View published research articles from *PLOS ONE* for specific examples.<br><br>This statement is required for submission and **will appear in the published article** if the submission is accepted. Please make sure it is accurate.	The author received no specific funding for this work.

Powered by Editorial Manager® and ProduXion Manager® from Aries Systems Corporation

**Unfunded studies**<br>Enter: *The author(s) received no specific funding for this work.*<br><br>**Funded studies**<br>Enter a statement with the following details:<br>• Initials of the authors who received each award<br>• Grant numbers awarded to each author<br>• The full name of each funder<br>• URL of each funder website<br>• Did the sponsors or funders play any role in the study design, data collection and analysis, decision to publish, or preparation of the manuscript?<br>• **NO** - Include this sentence at the end of your statement: *The funders had no role in study design, data collection and analysis, decision to publish, or preparation of the manuscript.*<br>• **YES** - Specify the role(s) played.<br><br>* typeset	
**Competing Interests**<br><br>Use the instructions below to enter a competing interest statement for this submission. On behalf of all authors, disclose any competing interests that could be perceived to bias this work—acknowledging all financial support and any other relevant financial or non-financial competing interests.<br><br>This statement is required for submission and **will appear in the published article** if the submission is accepted. Please make sure it is accurate and that any funding sources listed in your Funding Information later in the submission form are also declared in your Financial Disclosure statement.<br><br>View published research articles from *PLOS ONE* for specific examples.	NO authors have competing interests

**NO authors have competing interests**<br><br>Enter: *The authors have declared that no competing interests exist.*<br><br>**Authors with competing interests**<br><br>Enter competing interest details beginning with this statement:<br><br>*I have read the journal's policy and the authors of this manuscript have the following competing interests: [insert competing interests here]*<br><br>* typeset	
**Ethics Statement**<br><br>Enter an ethics statement for this submission. This statement is required if the study involved:<br><br>• Human participants<br>• Human specimens or tissue<br>• Vertebrate animals or cephalopods<br>• Vertebrate embryos or tissues<br>• Field research<br><br>Write "N/A" if the submission does not require an ethics statement.<br><br>General guidance is provided below. Consult the submission guidelines for detailed instructions. **Make sure that all information entered here is included in the Methods section of the manuscript.**	N/A

Powered by Editorial Manager® and ProduXion Manager® from Aries Systems Corporation

**Format for specific study types**<br><br>**Human Subject Research (involving human participants and/or tissue)**<br>• Give the name of the institutional review board or ethics committee that approved the study<br>• Include the approval number and/or a statement indicating approval of this research<br>• Indicate the form of consent obtained (written/oral) or the reason that consent was not obtained (e.g. the data were analyzed anonymously)<br><br>**Animal Research (involving vertebrate animals, embryos or tissues)**<br>• Provide the name of the Institutional Animal Care and Use Committee (IACUC) or other relevant ethics board that reviewed the study protocol, and indicate whether they approved this research or granted a formal waiver of ethical approval<br>• Include an approval number if one was obtained<br>• If the study involved *non-human primates*, add *additional details* about animal welfare and steps taken to ameliorate suffering<br>• If anesthesia, euthanasia, or any kind of animal sacrifice is part of the study, include briefly which substances and/or methods were applied<br><br>**Field Research**<br><br>Include the following details if this study involves the collection of plant, animal, or other materials from a natural setting:<br>• Field permit number<br>• Name of the institution or relevant body that granted permission	
**Data Availability**<br><br>Authors are required to make all data underlying the findings described fully available, without restriction, and from the time of publication. PLOS allows rare exceptions to address legal and ethical concerns. See the PLOS Data Policy and FAQ for detailed information.	Yes - all data are fully available without restriction

Powered by Editorial Manager® and ProduXion Manager® from Aries Systems Corporation

A Data Availability Statement describing where the data can be found is required at submission. Your answers to this question constitute the Data Availability Statement and **will be published in the article**, if accepted.<br><br>**Important:** Stating 'data available on request from the author' is not sufficient. If your data are only available upon request, select 'No' for the first question and explain your exceptional situation in the text box.<br><br>Do the authors confirm that all data underlying the findings described in their manuscript are fully available without restriction?	
**Describe where the data may be found in full sentences. If you are copying our sample text, replace any instances of XXX with the appropriate details.**<br><br>• If the data are **held or will be held in a public repository**, include URLs, accession numbers or DOIs. If this information will only be available after acceptance, indicate this by ticking the box below. For example: *All XXX files are available from the XXX database (accession number(s) XXX, XXX.).*<br>• If the data are all contained **within the manuscript and/or Supporting Information files**, enter the following: *All relevant data are within the manuscript and its Supporting Information files.*<br>• If neither of these applies but you are able to provide **details of access elsewhere**, with or without limitations, please do so. For example:<br><br>*Data cannot be shared publicly because of [XXX]. Data are available from the XXX Institutional Data Access / Ethics Committee (contact via XXX) for researchers who meet the criteria for access to confidential data.*<br><br>*The data underlying the results presented in the study are available from (include the name of the third party*	All relevant data are within the manuscript and its Supporting Information files.

and contact information or URL).<br>• This text is appropriate if the data are owned by a third party and authors do not have permission to share the data.<br><br>* typeset	
Additional data availability information:	

Powered by Editorial Manager® and ProduXion Manager® from Aries Systems Corporation

Manuscript

Click here to access/download;Manuscript;GC-MS analysis of fatty acids .docx

GC-MS analysis of fatty acids in healthy and *Aspergillus niger* MH078571.1-infected Arabica coffee beans.

Amira H. Alabdalall[1,2*]

[1]Department of Biology, College of Science, Imam Abdulrahman Bin Faisal University, P.O.Box 1982, Dammam, Saudi Arabia.

[2]Basic and Applied Scientific Research Center (BASRC), Imam Abdulrahman Bin Faisal University, P.O. Box 1982, Dammam 31441, Saudi Arabia.

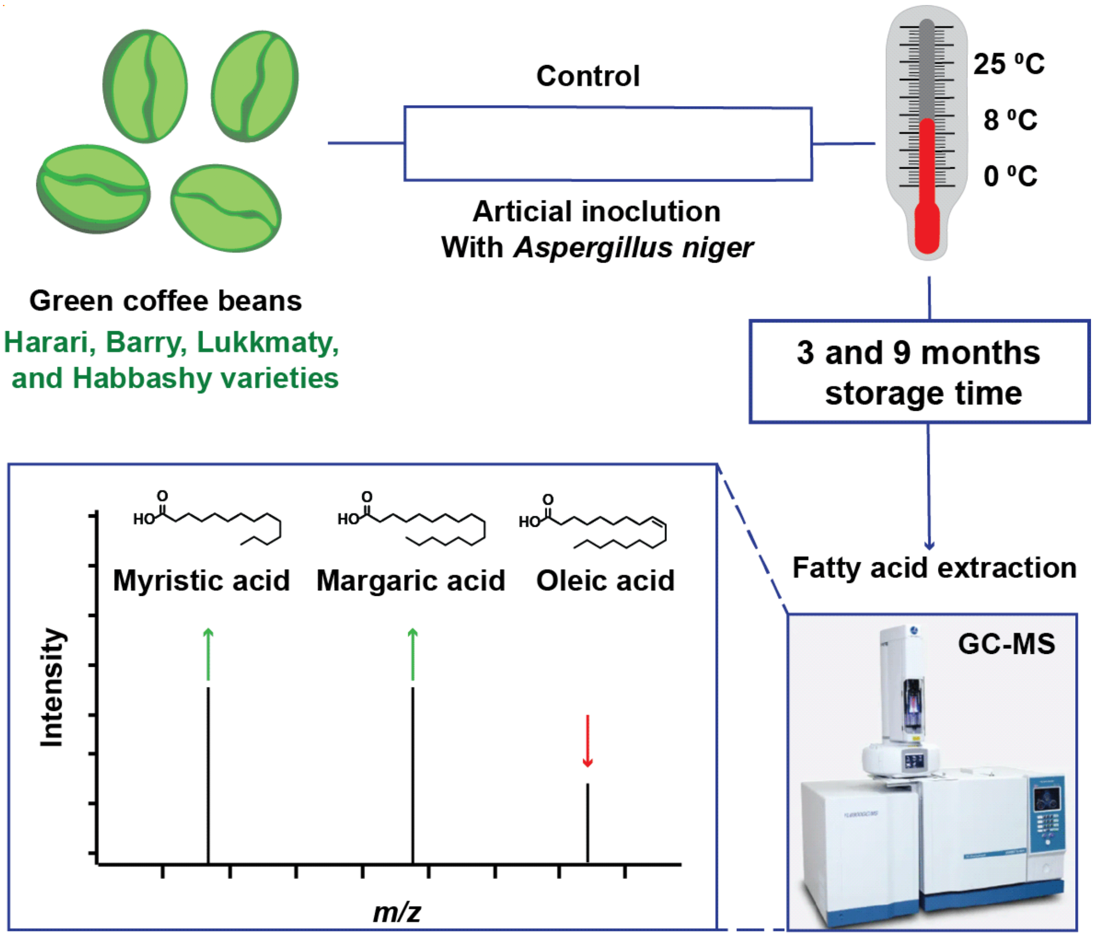

Graphical Abstract

ABSTRACT

The organic composition of Arabica coffee beans, particularly fatty acids, significantly influences their overall quality. After measuring its composition of fatty acids, it contained a high percentage of saturated fatty acids, including caprylic, lauric, myristic, palmitic, margaric, fat, and orchid. Moreover, the sample contained unsaturated fatty acids, namely palmitoleic acid (C16:1), oleic acid (C18:1), linoleic acid (C18:2), and alpha-linoleic acid (C18:3).

Coffee beans are susceptible to infection by fungi during storage, the development of which has adverse effects on the beans. The present study aimed to examine the impact of *Aspergillus niger* MH078571.1 infection on the diversity and abundance of fatty acids in green Arabica coffee beans. The impact of *Aspergillus niger* on the consumption of fatty acids in Arabica coffee beans was assessed. The findings of the study indicate that the duration of storage had a significant impact on the levels of fatty acids, specifically miristic (C14:0), margaric (C17:0), and stearic (C18:0), which increased as the storage period and temperature increased. Conversely, the percentage of oleic acid decreased under these conditions. This trend was observed across different storage temperatures (0, 8, and 25 °C) in untreated coffee beans affected by fungal activity.

Keywords: Coffee beans; fatty acid; margaric; stearic; oleic; miristic; *Aspergillus niger*.

INTRODUCTION

Mekete [1] indicated that coffee has a history; it has been known since the beginning of the ninth century B.C. Coffee was transported from Ethiopia to Egypt and Yemen. In the fifteenth century, it reached Italy, Europe, and America from the Islamic world and eventually reached Azerbaijan, Turkey, and Africa. Coffee is a widely consumed beverage all across the world. The majority of individuals drink coffee every day [1-3]. According to some surveys, it is the world's second most traded commodity after oil [4]. Around 103 species of coffee are known globally, 70 of which are produced in the tropics, and only three are farmed for commercial use Coffea arabica, Coffea robusta, and Coffea liberica [5,6].

Arabica coffee is distinct from Robusta coffee in several respects, including fragrance, taste, and stimulating effect [7-9]. Arabica coffee accounts for 64% of world coffee output and is extensively watched due to its remarkable organoleptic properties. Robusta coffee accounts for 35% of worldwide labor, has less flavor, and is more bitter than Arabica coffee. Robusta coffee is a less expensive alternative to Arabica coffee, while Liberica coffee is far less popular and accounts for substantially less worldwide output. Commercially important species include both Robusta and Arabica [5]. They are essential commodities in international trade, and only the best quality is acceptable [10].

The chemical components contained within fresh beans impact coffee's sensory properties [11]. The chemical content and quality of coffee beans vary significantly depending on the coffee variety, height, soil, daily temperature changes, and growing area [12-16]. Because of the multiple volatile and nonvolatile chemical components, coffee tastes vary. Coffee quality is affected by pre-harvest, post-harvest, and export handling. Other potential hazards to the coffee business include climate

change and plant disease[17]. Figueiredo et al.[18] assessed fatty acid levels in coffee beverages by gathering data on the primary compounds that exert either positive or negative influences on their quality. The researchers placed emphasis on the potential of saturated fatty acids, such as arachidic, stearic, and palmitic acids, to serve as discriminators for assessing the quality of specialty coffees. These fatty acids are indicative of superior sensory quality. On the other hand, it is worth noting that unsaturated fatty acids, such as elaidic, oleic, linoleic, and linolenic acid, have been found to potentially contribute to the characteristics of coffees with reduced acidity, fragrance, body, and flavor.

Fatty acids serve as the principal components of lipids. Triacylglycerols, typically observed in the form of three fatty acids bonded to a glycerol molecule, are commonly encountered. The properties of the glycerol molecule, including its digestibility and melting point, are influenced by the presence of various fatty acids within it [19]. Fatty acids are fats found in all living organisms. It is the most essential component of coffee beans, and green Arabica coffee beans contain a high concentration of fatty acids [8, 20, 21]. Most consist of triglycerides, sterols, and tocopherols [22]. The oil obtained from the green Arabica coffee beans is characterized by a significant abundance of fatty acids [8,21]. Martin et al.[21] established that green coffee beans comprise linoleic and palmitic acids as the primary fatty acids, alongside secondary acids such as myristic, palmitoleic, eicosinoic, behenic, linolenic, and arachidic acids. The acids in question exist in three distinct forms. These forms include saturated fatty acids, which are present in coffee oil within the range of 49.4-59.2%. Additionally, monounsaturated fatty acids are found in the range of 30-9.69%, while polyunsaturated fatty acids are present within the range of 29.5-39.2% [23, 24].

Coffee oil possesses beneficial properties as an emollient, skin therapy, and essential component in the human diet due to its involvement in prostaglandin synthesis and various biological processes associated with cell regeneration. The lack of coffee oil has been linked to the occurrence of skin issues [24]. According to Romano et al. [8], coffee oil is characterized by a significant abundance of palmitic acid, a compound known for its exceptional ability to safeguard the skin. Green coffee beans contain high levels of linoleic and palmitic acids.

Fatty acids are essential in affecting coffee quality. It is impacted by altitude, soil, and coffee plant position. As a result, the acid comparison is utilized as a classification approach, and the concentration of fatty acids is determined by the components distinguishing between coffee varieties and origins [21, 25]. Many investigations on these factors and their impact on the quantity and types of fatty acids have been carried out [12, 26-30]. Still, few studies on the influence of fungal molds on fatty acids have been conducted. Coffee beans are fermented in 10 cm thick layers and dried in the sun for 10-25 days. Approximately 754 microbial isolates are fermented by cellulose and pectin lysis; these fermentations impact coffee quality and shelf life [31]. Betancourt and Frank [32] suggested a humidity below 14.5% to avoid mold growth during storage.

Despite the recognized significance of fatty acids in contributing to the flavor and aroma of coffee, there is a lack of research investigating the impact of fungal molds on the composition and characteristics of fatty acids in coffee beans. As a result, this study aimed to determine how storage duration, temperature, and fungal infection affected fatty acid levels in the local market's most popular

green coffee beans.

MATERIALS AND METHODS

Sample collection

Green coffee bean samples of the four major coffee bean varieties, Harari, Barry, lukkmaty, and habbashy, were obtained from local marketplaces in Dammam and Al-Khobar, in the Eastern Province of the Kingdom of Saudi Arabia.

Preparing coffee beans for fungus infection:

The study used healthy coffee beans, which were chosen from four different varieties. These beans were carefully selected to ensure that they did not exhibit any defects. Each variety was then divided into two groups: one group served as the control, while the other group was subjected to fungus infection. To ensure sterilization, Arnold's sterilizer was employed. The calculation of relative humidity was performed. After sterilization, the humidity was adjusted to 10% for all samples, both the control group and the other group that was industrially contaminated with *Aspergillus niger* MH078571.1, and this strain was previously identified as the highest lipase-producing *Aspergillus niger* strain [33]. Three replicates were prepared for each treatment and incubated at 0, 8, and 25 °C for 3 and 9 months.

Bean fatty acid estimation:

Brockerhoff [34] using GLC to identify fatty acids in coffee bean hexane extracts:

Fatty acid extraction/methylation:

The coffee oil obtained through hexane extraction. The solvent was subjected to reduced pressure, resulting in its evaporation. The remaining substance was then subjected to saponification in a boiling water bath for a duration of 30 minutes. This process involved the use of an alcoholic solution of sodium hydroxide with a concentration of 15% w/v. Following the cooling process, a volume of 20 mL was subsequently transferred into a separatory funnel. The mixture underwent vigorous agitation subsequent to the addition of petroleum ether (60-80°C). The aqueous layer was acidified using a 1:1 ratio of hydrochloric acid (HCl). The free fatty acids were subjected to a triple extraction process using petroleum ether (60-80°C) in 10 mLaliquots. The solvents were subsequently

recycled by rinsing them with distilled water until the pH indicator phenolphthalein reached a neutral state. Following the removal of moisture using anhydrous sodium sulfate, the petroleum ether extracts were subjected to evaporation under reduced pressure. Dissolving fatty acids (1-2 mg) in anhydrous diethyl ether (0.5:1 mL), we then added diazomethane solution drop by drop until the yellow hue was maintained and the nitrogen gas bubbles ended. In 15 minutes at room temperature, the solvent was gone. The last step included dissolving fatty acid methyl esters in chloroform so that they could be detected in aliquots using gas-liquid chromatography.

Gas-liquid chromatography of fatty acid methyl esters:

For the gas chromatography with the flame ionization detector and the 1.5 by 4 mm coiled glass column with the 10% PEGA supported by the acid washed diatomic C (100-120 mesh), the GCV pye Unicam was used. The sample was injected into the column using Hamilton micro syringes. Column temperature in isothermal gas chromatography is 190 degrees Celsius, detector temperature is 300 degrees Celsius, and injection temperature is 250 degrees Celsius. Airflow of 330 mL min-1, hydrogen flow of 33 mL min-1, and nitrogen flow of 30 mL min-1. Average chart speed is 1 cm every 2 minutes, with a precision of 32×10^{-2}.

Identification and quantification:

Accurate materials, such as methyl esters of 10:0-20:0 fatty acids, were employed to determine the retention times of new fatty acids. The Philips PU 4810 computer integrator was used for peak detection and quantification.

Statistical analysis

Using SPSS version 23 [35], we performed an analysis of variance (ANOVA) to look for correlations between the control parameters and fungal infection.

RESULTS

The composition of coffee oil in all four varieties examined consisted of both saturated and unsaturated fatty acids at varying levels, which were specific to each coffee variety. The saturated fatty acids present included Caprilic acid (C10:0), Laurie acid (C12:0), Miristic acid (C14:0), Palmitic acid (C16:0), Margaric acid (C17:0), Stearic acid (C18:0), Arachidic acid (C20:0), and Eicosenoic acid (C22:0). Additionally, the unsaturated fatty acids

detected were Palmitoleic acid (C16:1), Oleic acid (C18:1), Linoleic acid (C18:2), and Alpha-Linoleic acid (C18:3).

The present study investigated the impact of fungal infection and storage conditions on the composition of fatty acids in coffee beans. Our findings indicate that both control and treated beans exhibited the presence of saturated and unsaturated fatty acid groups. The data presented in Tables S1-S8 indicate that miristic, margaric, stearic, oleic, and alpha-linoleic acids exhibited the highest frequencies, while the remaining types displayed negligible proportions (3%). The fatty acid composition was found to be affected by the duration of storage or the presence of fungal contamination. Fluctuations in temperatures were found to induce alterations in acid percentages. The findings indicated that the constituents of the bean were influenced by varying durations of storage. The fatty acid composition, specifically the presence of Oleic acid, in control samples of Harari, Barry, Lukkmaty, and Habashy beans, was significantly influenced by the storage temperature. After a period of 3 months at a temperature of 25OC, the respective percentages of Oleic acid were recorded as 42.97%, 41.67%, 42.56%, and 41.86%. Subsequently, after a duration of 9 months, the percentages of Oleic acid for the same bean varieties were observed as 13.76%, 24.26%, 19.76%, and 27.02%. The degradation of the samples infected with Aspergillus niger was observed to intensify as the percentages of Oleic fatty acids decreased. Specifically, after a period of 3 months, the percentages of Oleic fatty acids reached values of 40.29%, 39.53%, 41.76%, and 38.65% respectively under the same conditions. Furthermore, a significant reduction in oleic fatty acid content was observed in all tested varieties after 9 months, with percentages recorded as 10.15%, 17.02%, 18.79%, and 22.24% respectively.

The impact of storage duration on the fatty acid composition was observed in the four tested varieties of green coffee beans, namely Harari, Barry, Lukkmaty, and Habbashy. These varieties exhibited significant levels of saturated and unsaturated fatty acids, which were found to be influenced by both the storage period and temperature. The findings of the study indicate that the Habbashy variety exhibited a higher proportion of saturated acids, specifically 82.65%, when subjected to a temperature of 0°C for a period of 9 months. In comparison, the Lukkmaty variety displayed a slightly lower percentage of saturated acids at 81.54% under identical conditions. At a temperature of 8°C, the fatty acid compositions for the Barry and Harari cultivars were found to be 77.74% and 77.12%, respectively, as indicated in **Table 1**.

The results in **Table (1)** and **Figure (1)** show that the four studied varieties contained saturated and unsaturated fatty acids in different proportions, and it was noted that the total unsaturated fatty acids decreased to less than half in samples stored for 9 months compared to 3 months in all varieties.

Table 1: Total saturated and unsaturated fatty acids of the four types of green coffee beans after 3 and 9 months of artificial infection with *Aspergillus niger*.

Fatty acids	Varaity/period	control			A. niger		
		0°	8°	25°	0°	8°	25°
SFA	Harari/ 3 months	52.51	53.45	52.53	55.9	56.56	55.09
USFA		46.68	43.87	46.28	44.09	43.46	43.77
TFA		99.19	97.32	98.81	99.99	98.92	98.86
SFA	Harari/ 9 months	77.34	63.53	73.98	70.2	70.51	76.34
USFA		20.76	36.44	25.56	22.65	22.19	22.82
TFA		98.1	99.97	99.54	92.85	92.7	99.16
SFA	Barry / 3 months	54.05	54.1	64.61	56.23	53.59	60.3
USFA		45.9	45.83	35.32	43.53	46.31	39.6
TFA		99.95	99.93	99.93	99.76	99.9	99.9
SFA	Barry / 9 months	71.61	77.12	70	78.23	60.64	71.94
USFA		28.32	22.83	29.95	21.42	39.3	27.64
TFA		99.93	99.95	99.95	99.65	99.94	99.58
SFA	Lukkmaty / 3 months	53.69	58.09	54.46	54.52	64.7	63.32
USFA		46.25	41.95	45.5	45.32	35.21	36.57
TFA		99.94	99.96	99.96	99.84	99.91	99.89
SFA	Lukkmaty / 9 months	76.53	79.08	81.54	77.42	79.86	80.97
USFA		23.41	21.68	18.41	22.7	20.02	18.95
TFA		99.94	99.98	99.95	99.62	99.88	99.92
SFA	Habbashy / 3 months	54.41	55.56	55.7	57.52	57.27	53.92
USFA		45.55	44.37	44.25	42.32	42.69	46.06
TFA		99.96	99.93	99.95	99.84	99.96	99.98
SFA	Habbashy / 9 months	70.34	75.07	82.65	73.86	75.4	80.58
USFA		29.63	24.76	17.32	26.09	23.47	15.66
TFA		99.97	99.83	99.97	99.95	98.87	96.24

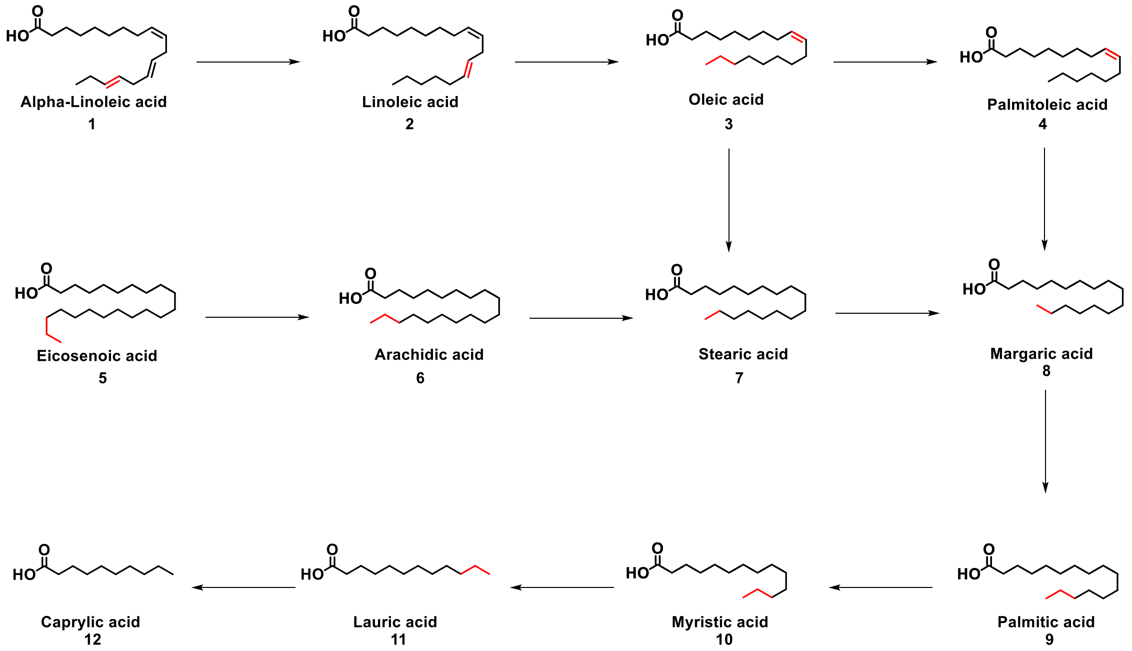

Figure 1 The proposed mechanism of fatty acid breakdown involves hydrogenation, which converts unsaturated fatty acid into saturated fatty acid.

In contrast, the saturated acids increased, and the results showed that this decrease and increase were greater in samples treated with *Aspergillus niger* **(Figures 2).**
Artificial fungal inoculation of beans revealed fungal infection of beans destroyed fatty acids, especially unsaturated ones. After 9 months at a temperature of 25°C, it decreased to 22.82, 27.64, 18.95, and 15.66% for Harari, Barry, Lukkmaty, and Habbashy cultivars, respectively **(Table 1)**.

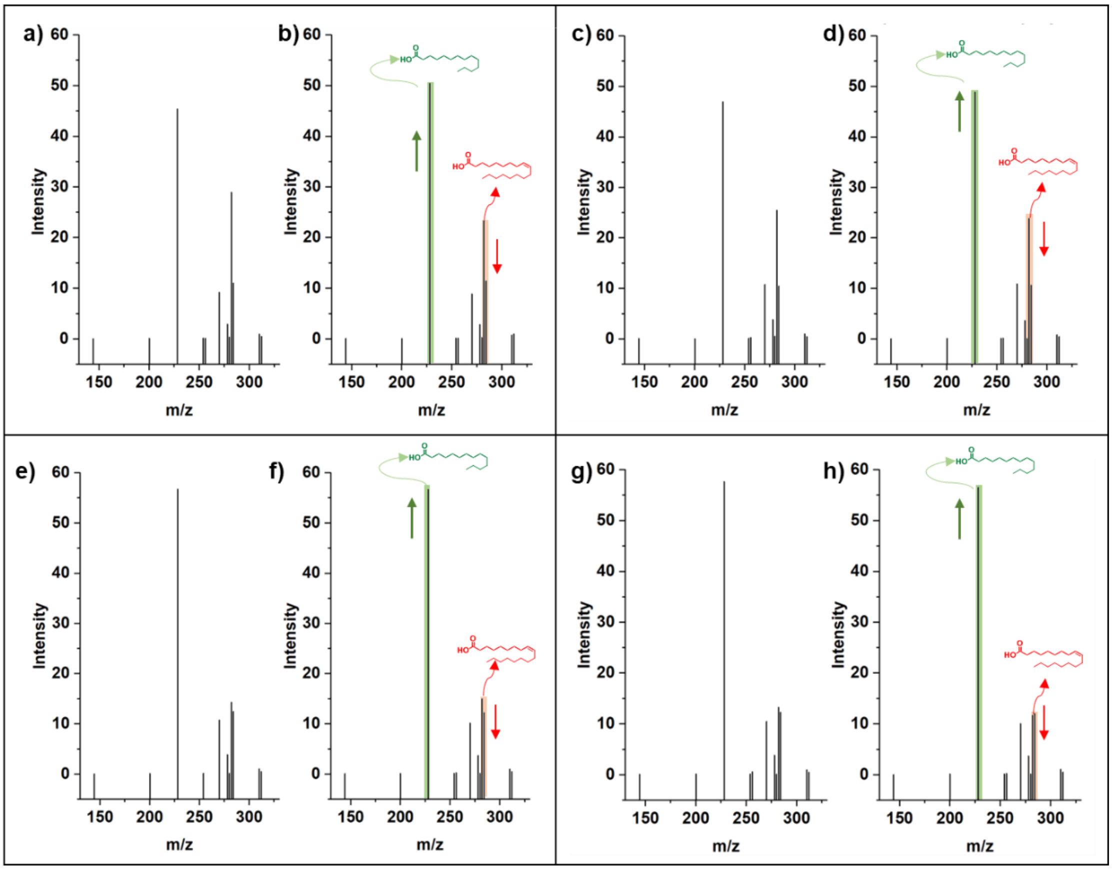

Figure 2 displays the chromatogram depicting the composition of fatty acids found in a sample of Arabica coffee beans. a) Harari control, b) Harari *A. niger*, c) Barry control, d) Barry *A. niger,* e) Lukkmaty control, f) Lukkmaty *A. niger*, g) Habbashy control, and h) Habbashy *A. niger***.**

DISCUSSION

The findings of the study indicated that the concentrations of saturated fatty acids (SFA), including stearic, margaric, and meristic acid, exhibited an upward trend following infection and prolonged storage durations, and elevated temperatures across all varieties of coffee beans. The levels of these fatty acids varied depending on the specific coffee variety being studied. The green coffee bean was found to contain two predominant acids, namely miristic acid and oleic acid. [36-40]. In a similar vein, it was observed that unsaturated fatty acids (USFA) exhibited a reduction in quantity because of both infection and storage, akin to oleic acid. These phenomena could potentially arise from the transfer of fatty acids to other molecules or from one variety to another. This finding was consistent with the findings of various researchers [36- 39,41,42].

The presence of infection in beans resulted in a greater reduction of oleic fatty acid compared to the control samples across all treated groups. This decrease was observed to be more pronounced as the storage period increased, likely due to the inherent propensity of oleic fatty acid to undergo reactions and convert into other fatty

acids [43, 44]. Furthermore, it was observed that the concentrations of miristic, margaric, and stearic acids, as well as fatty acids, exhibited an upward trend in infected beans as the storage period progressed. This phenomenon can potentially be attributed to the heightened hydrolytic activity of certain enzymes, such as lipase, and the subsequent degradation of fatty acids [44]. Additionally, it was noted that the functionality of lipase enzymes in sunflowers is influenced by both the temperature and duration of storage. In their study, Ghasemnez et al. [43] found similar outcomes for evening primrose as those reported by [45] when examining soybeans. Research has revealed that unsaturated fatty acids, including oleic, palmetiolic, linoleic, and alpha-linoleic acids, undergo rapid oxidation when exposed to oxygen. Consequently, an extended duration of storage is associated with a corresponding elevation in the rate of oxidation, and conversely. According to [46], a number of enzymes that play a role in the metabolic process of fatty acids, including acyl oxidase, malate synthase, and citrate synthase, are dependent on the presence of oxygen for their proper functioning. According to [44], variations in temperature have distinct impacts on the levels of fatty acids found in coffee beans.

The experimental conditions employed in this study promote the proliferation of the fungus and its enzymatic functions, leading to the degradation of seed tissues and subsequent deterioration. These findings align with previous research ~~conducted by~~ [47-49], which demonstrated a direct correlation between the decomposition of fava beans and factors such as humidity, temperature, and duration of storage. Additionally, the study conducted by [50,51] observed an augmentation in tissue pigmentation in maize grains when exposed to mildews. In their seminal work, Cole and Milner [52] expounded upon the phenomenon of bean coloration, attributing it to a chemical reaction involving nitrogenous compounds and reducing sugars. According to [53], the presence of brown coloration in the inner tissues can be attributed to the process of fatty acid oxidation. The quality of coffee and its flavor components have been attributed to the composition of fatty acids present in the coffee, as stated by [18] in their 2015 study. In a study conducted by [54], it was observed that bacteria and fungi are capable of synthesizing multiple enzymes involved in cell wall metabolism. These enzymes are responsible for cleaving the methyl ester bonds found in ferulic and coumaric acids, thereby enhancing the activity of hemicellulose enzymes during the hydrolysis process of cinnamic acid [55].

Several studies have highlighted the impact of storage duration, humidity, and temperature on product quality, including the works of [54, 55]. Several studies have indicated that the oil content in sunflowers tends to decrease after a storage period of three months. [56, 57, 58] have all reported this phenomenon. The decrease in oil content can be attributed to the combined effects of elevated temperature and humidity during storage, which promote oil decomposition and lead to an increase in free fatty

acids. In a study conducted by [59], the fatty acid levels of two coffee samples were examined. These samples were vacuum-packed and stored at a temperature of 25 °C for a duration of 180 days. The first sample consisted of pure Arabica beans, while the second sample was a blend of 80% Arabica and 20% Robusta beans. The findings of the study revealed that only the second sample exhibited elevated levels of fatty acids. In their study, Ghasemnez and Honermeier [44] documented a reduction in the levels of oleic acid from 90.6% to 88.2% over a period of 4 months during storage. They attributed this decline to the acid's propensity to undergo reactions and transition into free forms. A parallel effect of palmalic and linoleic acids has been identified. In their study, Ghasemnez and Honermeier [44] observed a reduction in the concentration of oleic acid from 90.6% to 88.2% following a storage period of 4 months. The authors attributed this decline to the activity of enzymes, specifically lipase. The explanation provided elucidates the acid's capacity to engage in interactions and transitions towards liberated states, a phenomenon that is similarly observed in palmalic and linoleic acids.

Moon et al,[40] investigated the causes of coffee losing its quality and distinct flavor. They confirmed that the oxidation processes that occur to fatty acids for example, when kept at high temperatures and for more extended storage periods, lead to increased oxidation processes in fats and a loss of taste quality. The first stage in tasting coffee is Olfaction since the aroma is perceived before the flavor. Coffee odor is attributed to volatile compounds in the brew activating neurons in the nasal canal. Caffeine, trigonelline, chlorogenic acid, and volatile compounds such as terpenoids are all components of a well-balanced cup of coffee [60].

According to the findings of Shahidi and Hossain [61], the process of oxidation of unsaturated fatty acids leads to the formation of hydroperoxides, which subsequently undergo decomposition to produce volatile secondary lipid oxidation products such as aldehydes, alcohols, and ketones, which are known to contribute to the development of odor.

Unsaturated fatty acids (USFA), including Palmitic, Stearic, and Alpha-Linoleic acids, were found in higher concentrations than saturated fatty acids (SFA). During storage, the amount of USFA dropped while the quantity of SFA increased.

Longer periods of storage resulted in a decrease in Stearic, Oleic, and Linoleic acid due to oxidation, whereas shorter periods of storage increased Palmitic and Arachidic acid. Acidity, aroma, body, and flavor all become less evident with greater USFA levels. Therefore, the high level of SFA in specialty coffees, especially palmitic, stearic, and arachidic acids [18, 62], contribute to their distinctive sensory character. However, the drink takes on a disagreeable taste and odor owing to the high quantities of polyunsaturated fatty acids, which is caused by the simple breakdown of double bonds and the creation of foul-flavored chemicals [63].

CONCLUSION

~~The four most common Arabica green coffee bean cultivars in the eastern area were subjected to experiments examining the impact of fungal infection with *A. niger* MH078571.1, as well as the impact of storage times and temperatures on the fatty acid content of green beans.~~ All of the cultivars' grains were found to have various concentrations of 11 distinct saturated and unsaturated fatty acids. At the conclusion of the storage time, oleic acid was found to have decreased drastically, whereas Miristic, margaric, and stearic acids had increased; further degradation was seen as a result of fungal infection.

Data Availability

Data used to support the findings of this study are included within the article.

Conflicts of Interest

The author declares that there are no conflicts of interest.

Acknowledgments

The author would like to acknowledge Dr. Fatimah A. Aldakheel- Polymer Synthesis Laboratory, KAUST Catalysis Center, Physical Sciences and Engineering Division, King Abdullah University of Science and Technology (KAUST) for sharing her knowledge and experience.

Funding This research received no external funding.

Declarations

Conflict of interest The author declare no conflict of interest.

References

1. Mekete B. Coffee, in van Uhlig. In, *Encyclopaedia Aethiopica, Siegbert* (Ed.). Horrowitz, Weissbaden, **2003**, Pages 763.
2. Yisak H.; Redi-Abshiro M.; Chandravanshi B.S. New fluorescence spectroscopic method for the determination of alkaloids in green coffee beans.*Chem Cent J* **2018a**,*12*, 59. doi.org/10.1186/s1306 5-018-0431-4.
3. Yisak H.; Redi-Abshiro M.; Chandravanshi B.S. Selective determination of caffeine and trigonelline in aqueous extract of green coffee beans by FT-MIR-ATR spectroscopy. Vib Spectrosc, 2018b, 97, 33–38. DOI:10.1016/J.VIBSPEC.2018.05.003
4. Mussatto S.I.; Machado E.M.S.; Martins S.; Teixeira J.A. Production, composition, and application of coffee and its industrial residues. Food Bioprocess Technol 2011, 4, 661–672. https://doi.org/10.1007/s11947-011-0565-z

5. Pohlan H.A.J.; Janssens M.J.J. Growth and production of coffee. In, Verheye WH, editor. Soils, plant growth and crop production. Encyclopedia of Life Support System (EOLSS), Developed under the Auspices of the UNESCO, Eolss Publishers, Oxford, UK, 2010. [http, //www.eolss.net] [Retrieved December 4, 2010

6. Davis A.P.; Govaerts R.; Bridson D.M.; Stoffelen P. An annotatated taxonomic of the genus *Coffea* (Rubiaceae). *Bot J Linnean Soc* 2006, 152, 465–512. https://doi.org/10.1111/j.1095-8339.2006.00584.x

7. Casal S.; Oliveira M.B.P.P.; Alves M.R.; Ferreira M.A. Discriminate analysis of roasted coffee varieties for trigonelline, nicotinic acid, and caffeine content. *J Agric Food Chem* 2000, 48, 3420–3424. https:// DOI: 10.1021/jf990702b

8. Romano R.; Santini A.; Grottaglie L.L.; Manzo N.; Visconti A.; Ritieni A. Identification markers based on fatty acid composition to differentiate between roasted *Arabica* and *Canephora* (*Robusta*) coffee varieties in mixtures. *J Food Compos Anal* **2014**, *35*, 1–9. https://DOI:10.1016/j.jfca.2014.04.001

9. Gebeyehu B.T.; Bikila S.L. Determination of caffeine content and antioxidant activity of coffee. *Am J Appl Chem* **2015**, *3*, 69–76. doi: 10.11648/j.ajac.20150302.16

10. Huck C.W.; Guggenbichler W.; Bonn G.K. Analysis of caffeine, theobromine, and theophylline in coffee by near-infrared spectroscopy (NIRS) compared to high-performance liquid chromatography (HPLC) coupled to mass spectrometry. *Anal Chim Acta* 2005, *538*, 195–203. DOI: 10.1016/j.aca.2005.01.064

11. Fassio L.O.; Malta M.R.; Carvalho G.R.; Liska G.R.; Lima P.M.; Nadaleti D.H.S.; Fonseca A.J.; Pimenta C.J. Fatty acids profile of *Coffea arabica* L. resistant to leaf rust grown in two environments of Minas Gerais, Brazil. *J Agric Sci* **2017**, *9*, 88–98. **DOI:**10.5539/jas.v9n12p88

12. Bertrand B.; Vaast P.; Alpizar E.; Etienne H.; Davrieux F.; Charmetant P. Comparison of bean biochemical composition and beverage quality of Arabica hybrids involving Sudanese-Ethiopian origins with traditional varieties at arious elevations in Central America. *Tree Physiol* **2006**, *26*, 1239–1248. DOI: 10.1093/treephys/26.9.1239

13. Villarreal D.; Laffargue A.; Posada H.; Bertrand B.; Lashermes P.; Dussert S. Genotypic and environmental effects on coffee (*Coffea Arabica* L.) bean fatty acid profile, impact on variety and origin chemometric determination. *J Agric Food Chem* **2009**, *57*, 11321–11327. DOI: 10.1021/jf902441n

14. Mehari B.; Redi-Abshiro M.; Chandravanshi B.S.; Combrinck S.; Atlabachew M.; McCrindle R. Profiling of phenolic compounds using UPLC–MS for

determining the geographical origin of green coffee beans from Ethiopia. *J Food Compos Anal* **2016a**, *45*, 16–25. DOI: 10.1016/j.jfca.2015.09.006

15. Mehari B.; Redi-Abshiro M.; Chandravanshi B.S.; Atlabachew M.; Combrinck S.; McCrindle R. Simultaneous determination of alkaloids in green coffee beans from Ethiopia, chemometric evaluation of geographical origin. *Food Anal Methods* **2016b**, *9*, 1627–1637. **DOI: 10.1007/s12161-015-0340-2**

16. Mehari B.; Redi-Abshiro M.; Chandravanshi B.S.; Combrinck S.; McCrindle R. Characterization of the cultivation region of Ethiopian coffee by elemental analysis. *Anal Lett* **2016c**, *49*, 2474–2489. **DOI:10.1080/00032719.2016.1151023**

17. Velásquez S. , Banchón C. Influence of pre-and post-harvest factors on the organoleptic and physicochemical quality of coffee, a short review. *J Food Sci Technol* **2022**, *5*, 1-13. **https://doi.org/10.1007/s13197-022-05569-z**

18. Figueiredo L.P.; Borém F.M.; Ribeiro F.C.; Giomo G.S.; Taveira J.H.S.; Malta M.R. Fatty acid profiles and parameters of quality of specialty coffees produced in different Brazilian regions. *African Journal of Agricultural Research* **2015**, *10* (35), 3484-3493. DOI, 10.5897/AJAR2015.9697.

19. Lunn J., Theobald H.E. The Health Effects of Dietary Unsaturated Fatty Acids. *Nutr Bull* **2006**, *31*, 178–224. doi, 10.1111/j.1467-3010.2006.00571.x.

20. Alvarez A.M.R.; Rodrguez M.L.G. Lipids in pharamaceutical and cosmetic preparations. Grasas Aceites 2000, 51, 74–96. https://doi.org/10.3989/gya.2000.v51.i1-2.409

21. Martn M.J.; Pablos F.; Gonzlez A.G.; Valdenebro M.S.; León-Camacho M. Fatty acid profiles as discriminant parameters for coffee varieties differentiation. *Talanta* **2001**, *54*, 291–297. DOI: 10.1016/s0039-9140(00)00647-0

22. Wagemaker T.A.L.; Carvalho C.R.L.; Maia N.B.; Baggio S.R.; Filho O.G. Sun protection factor, content, and composition of the lipid fraction of green coffee beans. *Ind Crops Prod* **2011**, *33*, 469–473. DOI: 10.1016/j.indcrop.2010.10.026

23. Dong W.; Hu R.; Chu Z.; Zhao J.; Tan L. Effect of different drying techniques on bioactive components, fatty acid composition, and volatile profile of robusta coffee beans. *Food Chem* **2017**, *234*, 121–130. DOI: 10.1016/j.foodchem.2017.04.156

24. Hurtado-Benavides A.; Dorado D.A.; Sánchez-Camargo A.D.P. Study of the fatty acid profile and the aroma composition of oil obtained from roasted Colombian coffee beans by supercritical fluid extraction. *J Supercrit Fluids* **2016**, *113*, 44–52. DOI: 10.1016/j.supflu.2016.03.008

25. Mehari B.; Redi-Abshiro M.; Chandravanshi B.S.; Combrinck S.; McCrindle R.; Atlabachew M. GC-MS profiling of fatty acids in green coffee (Coffea arabica L.) beans and chemometric modelling for tracing geographical origins from Ethiopia. *J Sci Food Agric* **2019**, *99*, 3811–3823. DOI: 10.1002/jsfa.9603

26. Sherge K.T.; D'heer J.; Duchateau L.; Boeckx P. Influence of growing altitude, shade and harvest period on quality and biochemical composition of Ethiopian specialty coffee. *J Sci Food Agric* **2017**, *97*, 2849–2857. DOI: 10.1002/jsfa.8114

27. Mintesnot A.; Dechassa N. Effect of altitude, shade, and processing methods on the quality and biochemical composition of green coffee beans in Ethiopia. *East Afr J Sci* **2018**, *12*, 87–100.

28. Adem M.W.; Meulenaer B.D.; Duchateau L.; Boeckx P. Effect of altitude on biochemical composition and quality of green arabica coffee beans can be affected by shade and postharvest processing method. *Food Res Int* **2018**, *105*, 278–285. DOI: 10.1016/j.foodres.2017.11.016

29. Bodner M.; Morozova K.; Kruathongsri P.; Thakeow P.; Scampicchio M. Effect of harvesting altitude, fermentation time and roasting degree on the aroma released by coffee powder monitored by proton transfer reaction mass spectrometry. *Eur Food Res Technol* **2019**, *245*, 1499–1506. **DOI :** 10.1007/s00217-019-03281-5

30. Hagos M.; Redi-Abshiro M.; Chandravanshi B.S.; Ele E.; Mohammed A.M.; Mamo H. Correlation between caffeine contents in green coffee beans and altitudes of the coffee plants grown in southwest Ethiopia using ultra violet visible spectrophotometry. *Bull Chem Soc Ethiop* **2018**, *32*, 13–25. DOI: 10.4314/bcse.v32i1.2

31. Silv C.F.; Scwan R.F.; Dias E.S.; Wheals A.E. Microbial diversity during maturation and natural processing of coffee cherries of coffee Arabica in Brazil. *Int. J. Food Microbial* **2000**, *30*, 251-260. DOI: 10.1016/s0168-1605(00)00315-9

32. Betancourt L.E.; Frank H.K. Behavior of ochratoxin A during green coffee roasting and soluble coffee manufacture. *J. Agric. Food Chem* **1983**, *46*, 673-675. DOI: 10.1021/jf9707703

33. Alabdalall A. H.; ALanazi N. A.; Aldakeel S. A.; AbdulAzeez S.; Borgio J. F. Molecular, physiological, and biochemical characterization of extracellular lipase production by *Aspergillus niger* using submerged fermentation. *Peer J* **2020**, *8*, e9425. https://doi.org/10.7717/peerj.9425

34. Brockerhoff, H. Stereo specific analysis oftriglyciedes. *J Lipid Res* **1965**,

6,10-15.
35. George D, Mallery P. IBM SPSS Statistics 23 step by step, a simple guide and reference, 14th ed.; New York Routledge.2016; Pages, 400. doi.org/10.4324/9781315545899

36. Sai A.M. Physicochemical Properties, and Aromatic Profile of Arabica Coffee in Ywangan Township, Myanmar. Master's Thesis, Mae Fah Luang University, Chiang Rai, Thailand, 2020.

37. Martın M.J.; Pablos F.; Gonzalez A.G.; Valdenebro M.S.; León-Camacho M. Fatty acid profiles as discriminant parameters for coffee varieties differentiation. *Talanta* **2001**, *54*, 291–297. DOI: 10.1016/s0039-9140(00)00647-0

38. Raba D.N.; Poiana M.-A.; Borozan A.B.; Stef M.; Radu F.; Popa M.-V. Investigation on Crude and High-Temperature Heated Coffee Oil by ATR-FTIR Spectroscopy along with Antioxidant and Antimicrobial Properties. *PLoS ONE* **2015**, *10*, e0138080. https://doi.org/10.1371/journal.pone.0138080

39. Vu D.C.; Vu Q.T.; Huynh L.; Lin C.-H.; Alvarez S.; Vo X.T.; Nguyen T.H.D. Evaluation of fatty acids, phenolics and bioactivities of spent coffee grounds prepared from Vietnamese coffee. *Int J Food Prop* **2021**, *24*, 1548–1558. https://doi.org/10.1080/10942912.2021.1977657

40. Moon A.S.; Wongsakul S.; Kitazawa H.; Saengrayap R. Lipid Oxidation Changes of Arabica Green Coffee Beans during Accelerated Storage with Different Packaging Types. *Foods* **2022**, *11*, 3040. https://doi.org/10.3390/foods11193040

41. Ahmad Khan M.; Shahidi F. Oxidative stability of stripped and no stripped borage and evening primrose oils and their emulsions in water. *J. Am Oil Chem Soc* **2000**, *77*, 963-968. https://doi.org/10.1007/s11746-000-0152-z

42. Muangkaeo, R., Srichuwong, S. Pawelzik, E. Verasilp, S. Influence of packaging material and storage time on seed viability and chemical component of rice seed. Proceedings of the Global Food and Product Chain Dynamics, Innovations, Conflicts, Strategies, act. 11-13, Deucher Tropentag, Hohenheim, pp, 1-5. 2005.
43. Ghasemnez had A.; Gergel S.; Honermeier B. The impact of storage time and storage temperature on the quality of the oil of evening primrose (*Oenothera biennis* L.). *J. Med Spice Plants* **2007**, *12*, 175-180.

44. Ghasemnez had A.; Honermeier B. Influence of storage conditions on quality and viability of high and low oleic sunflower seeds. *Int J Plant Prod* **2007**, *3*, 39-48.

45. Ulla Ullah J.; Hamayoun M.; Ahmad T.; Ayub M.; Zafarullah M. Effect of light, natural and synthetic antioxidants on stability of edible oil and fats. *Asian*

J. Plant Sci **2003**, *2*, 1192-1194.

46. Kindle, H. P-Oxidation of Fatty Acids by Specific Organelles. In, *The Biochemistry of Plants*. 9, Lipids, Structure and Function, Stumpf, P.K. and E.E. Conn (Eds.). Chapter 2, Harcourt Brace Jovanovich Publishing Company, San Diego, California, 1987; pp, 31-53.
47. Parenicova L.; Benen J.A.E.; Samson R.A.; Visser J. Evaluation of RFLP analysis of the classification of selected black Aspergilli. Myca!. *Res* **1997**, *101*, 810-814.
48. Lang C.; Dornenburg H. Perspectives in the biological function and the technological application of polygalacturonases. *Applied Microbial Biotechnol* **2000**, *53*, 366-375.
49. Silva C.F.; Batista L.R.; Abreu L.; Dias E.; Schwan R.F. Succession of bacterial and fungal communities during natural coffee (*Coffea arabica*) fermentation. *Food Microbiology* **2008**, *25*(8), 951-957. doi.org/10.1016/j.fm.2008.07.003

50. Batista L.R.; Chalffoun S.M.; Prado G.; Schwan R.F.; Wheals A.E. Toxigenic fungi associated with processed (green) coffee beans (*Coffea arabica* L.). *Int. J. Food Microbial* **2002**, *62*, 1-8.
51. Gerrit H.G. Enhancement of coffee quality by mould prevention. *Food Control* **2003**, *14*, 245-249.

52. Morello J.; Motilva M.; Tovar M.; Romero M. Changes in commercial virgin olive oil (cv. Arbequina) during storage, with special emphasis on the phenolic fraction. *Food Chem* **2004**, *85*, 357-364.

54. Sisman C.B.; Delibas L. Storing sunflower seed and quality losses during storage. *J Central Eur Agric* **2004**, *4*, 239-250. DOI: 10.2298/HEL0542115C
55. Sisman C.B. Quality losses in temporary sunflower seed stores and influences of storage conditions on quality losses during storage. *J Cent Eur Agric* **2005**, *6*, 143-150.

56. Villiers J.B.M.; Toit L.J.; Pretorius H.E. The be haviour of decorticated s unflower seeds stored under under favourable and unfavourable conditions. *Lebensrn- Wiss U- Technol* **1986**, *19*, 472-476.

57. Martini S.; Anon M.S. Storage of sunflower seed, Variation on the wax content of the oil. *Eur. J Lipid Sci Technol* **2005**, *107*, 74-79. DOI: 10.1002/ejlt.200401044

58. Neg S.C.; Anderson A. Lipid oxidation in Quinoa (Chenopodium quinoa) as determinated through accelerated aging. *J Environ Agric Food Chem* **2005**, *4*,

1010-1020.

59. Vila M.; Andueza S.; de Pena P.; Cid C. Fatty acid evolution during the storage of ground, roasted coffees. *JAocS* **2005**, *82*, 639-646. DOI: 10.1007/s11746-005-1122-1

60. Saloko S.; Sulastri Y.; MuradRinjani M. The effects of temperature and roasting time on the quality of ground *Robusta coffee* (*Coffea rabusta*) using Gene Cafe roaster. *Biosci Biotechnol Biomet* **2019**, DOI: 10.1063/1.5141310

61. Shahidi F.; Hossain A. Role of Lipids in Food Flavor Generation. *Molecules* **2022**, *27*(15), 5014. doi, 10.3390/molecules27155014. https://doi.org/10.3390/molecules27155014

62. Tsegay G.; Redi-Abshiro M.; Chandravanshi B.S.; Ele E.; Mohammed A.M.; Mamo H. Effect of altitude of coffee plants on the composition of fatty acids of green coffee beans. *BMC Chem* **2020**, *14*, 36. https://doi.org/10.1186/s13065-020-00688-0

63. Cong S.; Dong W.; Zhao J.; Hu R.; Long Y.; Chi X. Characterization of the Lipid Oxidation Process of Robusta Green Coffee Beans and Shelf-Life Prediction during Accelerated Storage. *Molecules* **2020**, *25*, 1157. https://doi.org/10.3390/molecules25051157

Click here to access/download
Supporting Information
Supplements data.docx
