# Supplementary material for: Gas chromatography–mass spectrometry analysis of fatty acids in healthy and Aspergillus niger MH078571.1-infected Arabica coffee beans
Source: PLoS One. 2024 Jan 2;19(1):e0293369. doi: 10.1371/journal.pone.0293369 (PMC10760658; doi:10.1371/journal.pone.0293369)
Supplement: Attachment — Submitted filename: Authors responses (GC).docx [file pone.0293369.s003.docx]

**Author’s response**

Manuscript Number: PONE-D-23-23915 

GC-MS analysis of fatty acids in healthy and *Aspergillus niger* MH078571.1-infected

Arabica coffee beans.

**Response to the notes of the first reviewer:**

I express my heartfelt gratitude to the first reviewer of the article, who diligently scrutinized its contents and provided several revisions, which were instantly included in the document. All the modifications have been thoroughly examined and authorized. I have compiled these questions and suggestions in the table provided below, along with I am corresponding responses to these modifications and recommendations:

| After correction | Before correction | Section | Page |
| --- | --- | --- | --- |
| [Gas chromatography–mass spectrometry](https://en.wikipedia.org/wiki/Gas_chromatography%E2%80%93mass_spectrometry)  analysis of fatty acids in healthy and *Aspergillus* *niger* MH078571.1-infected  Arabica coffee beans. | GC-MS analysis of fatty acids in healthy and *Aspergillus niger* MH078571.1-infected  Arabica coffee beans. | Title: It is not preferable to use abbreviations in the title for more clarity. | 1 |
| saturated fatty acids (SFA), unsaturated fatty acids (USFA), | saturated fatty acids (SFA), unsaturated fatty acids (USFA), | ABSTRACT | 1 |
| *Aspergillus niger;* Coffee beans; fatty acid; margaric; miristic; stearic; oleic | Coffee beans; fatty acid; margaric; stearic; oleic; miristic; *Aspergillus niger* | Keywords: Please rearrange key words alphabetically | 2 |
| Historically, coffee has been known since the beginning of the ninth century B.C. (1). | Mekete [1] indicated that coffee has a history; it has been known since the beginning of the ninth century B.C. | NTRODUCTION:  Too long, it should cover three parts: the background, the scientific significance, and the aim of the study. It is noted that there are repeated paragraphs or cover the same idea, so please shorten the introduction to include these parts for more clarity. | 2 |
| Coffee is consumed worldwide; most people consume coffee daily [1-3]. Some estimates say it's the second most traded commodity after oil [4]. Globally, there are 103 coffee species, 70 of which are tropical, and only three are commercially grown. Coffea arabica, robusta, liberica [5,6]. | The majority of individuals drink coffee every day [1-3]. According to some surveys, it is the world's second most traded commodity after oil [4]. Around 103 species of coffee are known globally, 70 of which are produced in the tropics, and only three are farmed for commercial use Coffea arabica, Coffea robusta, and Coffea liberica [5,6]. |  | 2 |
| Arabica coffee has a different aroma, flavor, and stimulating impact than Robusta [7-9]. Arabica coffee, which makes up 64% of global coffee production, is closely monitored for its organoleptic qualities. Robusta coffee accounts for 35% of global labor, has less taste, and is bitterer than Arabica. Liberica coffee is less popular and produces less globally than Robusta coffee, which is cheaper. Economically significant species are Robusta and Arabica [5]. International commerce relies on them, thus only the finest quality is acceptable [10]. | Arabica coffee is distinct from Robusta coffee in several respects, including fragrance, taste, and stimulating effect [7-9]. Arabica coffee accounts for 64% of world coffee output and is extensively watched due to its remarkable organoleptic properties. Robusta coffee accounts for 35% of worldwide labor, has less flavor, and is more bitter than Arabica coffee. Robusta coffee is a less expensive alternative to Arabica coffee, while Liberica coffee is far less popular and accounts for substantially less worldwide output. Commercially important species include both Robusta and Arabica [5]. They are essential commodities in international trade, and only the best quality is acceptable [10]. |  | 2 |
| Fresh beans' chemicals affect coffee's taste [11]. Coffee bean chemical composition and quality vary greatly by variety, height, soil, daily temperature variations, and growing location [12-16]. Variable volatile and nonvolatile chemical components make coffee taste different. Coffee quality is altered by pre-, post-, and export handling. Climate change and plant disease are further coffee industry risks[17]. | The chemical components contained within fresh beans impact coffee's sensory properties [11]. The chemical content and quality of coffee beans vary significantly depending on the coffee variety, height, soil, daily temperature changes, and growing area [12-16]. Because of the multiple volatile and nonvolatile chemical components, coffee tastes vary. Coffee quality is affected by pre-harvest, post-harvest, and export handling. Other potential hazards to the coffee business include climate change and plant disease[17]. |  | 2 |
| analyzed coffee's fatty acid content by identifying the main constituents that affect quality. The researchers focused on saturated fatty acids such arachidic, stearic, and palmitic acids as speciality coffee quality indicators. These fatty acids provide sensory excellence. However, unsaturated fatty acids including elaidic, oleic, linoleic, and linolenic acid may contribute to coffees with lower acidity, smell, body, and taste [18]. | Figueiredo et al.[18] assessed fatty acid levels in coffee beverages by gathering data on the primary compounds that exert either positive or negative influences on their quality. The researchers placed emphasis on the potential of saturated fatty acids, such as arachidic, stearic, and palmitic acids, to serve as discriminators for assessing the quality of specialty coffees. These fatty acids are indicative of superior sensory quality. On the other hand, it is worth noting that unsaturated fatty acids, such as elaidic, oleic, linoleic, and linolenic acid, have been found to potentially contribute to the characteristics of coffees with reduced acidity, fragrance, body, and flavor. |  | 3 |
| Lipids are mostly fatty acids. Triacylglycerols—three fatty acids linked to a glycerol molecule—are prevalent. Fatty acids affect glycerol's digestion and melting point [19]. All living things have fatty acids. It is the most important part of coffee beans, and green Arabica beans are abundant in fatty acids [8, 20, 21]. The majority are triglycerides, sterols, and tocopherols [22]. The oil from green Arabica coffee beans is rich in fatty acids [8,21]. Green coffee beans include main fatty acids like linoleic and palmitic acids and secondary acids like myristic, palmitoleic, eicosinoic, behenic, and arachidic acids, according to Martin et al.[21]. The acids have three forms. Coffee oil contains 49.4-59.2% saturated fatty acids. The range of monounsaturated fatty acids is 30-9.69%, whereas polyunsaturated is 29.5-39.2% [23, 24]. | Fatty acids serve as the principal components of lipids. Triacylglycerols, typically observed in the form of three fatty acids bonded to a glycerol molecule, are commonly encountered. The properties of the glycerol molecule, including its digestibility and melting point, are influenced by the presence of various fatty acids within it [19]. Fatty acids are fats found in all living organisms. It is the most essential component of coffee beans, and green Arabica coffee beans contain a high concentration of fatty acids [8, 20, 21]. Most consist of triglycerides, sterols, and tocopherols [22]. The oil obtained from the green Arabica coffee beans is characterized by a significant abundance of fatty acids [8,21]. Martin et al.[21] established that green coffee beans comprise linoleic and palmitic acids as the primary fatty acids, alongside secondary acids such as myristic, palmitoleic, eicosinoic, behenic, linolenic, and arachidic acids. The acids in question exist in three distinct forms. These forms include saturated fatty acids, which are present in coffee oil within the range of 49.4-59.2%. Additionally, monounsaturated fatty acids are found in the range of 30-9.69%, while polyunsaturated fatty acids are present within the range of 29.5-39.2% [23, 24]. |  | 3 |
| Coffee oil is an emollient, skin treatment, and vital dietary component owing to its role in prostaglandin formation and cell regeneration. Skin problems are associated to coffee oil deficiency [24]. Coffee oil contains a lot of palmitic acid, a skin-protecting ingredient. Green coffee beans are rich in linoleic and palmitic acids [8]. | Coffee oil possesses beneficial properties as an emollient, skin therapy, and essential component in the human diet due to its involvement in prostaglandin synthesis and various biological processes associated with cell regeneration. The lack of coffee oil has been linked to the occurrence of skin issues [24]. According to Romano et al. [8], coffee oil is characterized by a significant abundance of palmitic acid, a compound known for its exceptional ability to safeguard the skin. Green coffee beans contain high levels of linoleic and palmitic acids. |  | 3 |
| Coffee quality depends on fatty acids. It depends on height, soil, and coffee plant location. Thus, acid comparison is used to classify coffee kinds and origins, and fatty acid concentrations are determined by these components [21, 25]. Many studies have examined how these variables affect fatty acid amount and type [12, 26-30]. Few research have examined how fungal molds affect fatty acids. | Fatty acids are essential in affecting coffee quality. It is impacted by altitude, soil, and coffee plant position. As a result, the acid comparison is utilized as a classification approach, and the concentration of fatty acids is determined by the components distinguishing between coffee varieties and origins [21, 25]. Many investigations on these factors and their impact on the quantity and types of fatty acids have been carried out [12, 26-30]. Still, few studies on the influence of fungal molds on fatty acids have been conducted. |  | 3 |
| Coffee beans are fermented in 10 cm layers and sun-dried for 10-25 days. Cellulose and pectin lysis ferment 754 bacteria isolates, affecting coffee quality and shelf life [31]. lower humidity than 14.5% to prevent mold formation during storage [32]. | Coffee beans are fermented in 10 cm thick layers and dried in the sun for 10-25 days. Approximately 754 microbial isolates are fermented by cellulose and pectin lysis; these fermentations impact coffee quality and shelf life [31]. Betancourt and Frank [32] suggested a humidity below 14.5% to avoid mold growth during storage. |  | 3 |
| Done in the table. | • The findings are presented clearly, and the results are reliable. • Table 1: Clear and comprehensive. However, it is preferable to clarify the abbreviations in the lower margin of the table for more clarity. | RESULTS | 7 |
| The decrease in oil content can be attributed to the combined effects of elevated temperature and humidity during storage, which promote oil decomposition and lead to an increase in free fatty acids. Additionally, this phenomenon has been reported by several previous studies [56, 57, 58]. | [56, 57, 58] have all reported this phenomenon. The decrease in oil content can be attributed to the combined effects of elevated temperature and humidity during storage, which promote oil decomposition and lead to an increase in free fatty acids. | DISCUSSION:  • Please indicate the reference number immediately after the researchers' names, for example: Ulla et al. (45) and so on. | 10 |
| From the data obtained from this study, it has been concluded that all of the cultivars' grains were found to have various concentrations of 11 distinct saturated and unsaturated fatty acids. At the conclusion of the storage time, oleic acid was found to have decreased drastically, whereas Miristic, margaric, and stearic acids had increased; further degradation was seen as a result of fungal infection. | The four most common Arabica green coffee bean cultivars in the eastern area were subjected to experiments examining the impact of fungal infection with A. niger MH078571.1, as well as the impact of storage times and temperatures on the fatty acid content of green beans. All of the cultivars' grains were found to have various concentrations of 11 distinct saturated and unsaturated fatty acids. At the conclusion of the storage time, oleic acid was found to have decreased drastically, whereas Miristic, margaric, and stearic acids had increased; further degradation was seen as a result of fungal infection. | Conclusion:  It is preferable to focus on the conclusion of the study | 12 |

**Response to the notes of the second reviewer:**

I would like to express my gratitude and appreciation to the second reviewer for their diligent contribution in presenting several questions and ideas. I have compiled these queries and recommendations in the table below, along with our responses to these modifications and suggestions:

| After correction | Before correction | Section | Page |
| --- | --- | --- | --- |
| Due to their popularity and extensive use, local coffee types were highlighted. | -in the coffee varieties, you seem to include brands, you include the scientific name of the variety, composition, country of origin, there is no need for brands | ABSTRACT | 1 |
| The first reviewer noted this, which has been shortened. | - all the manuscript are very lengthy and not summarized making it hard to read by the reader. For example a very lengthy introduction isn't required and out of scope, no need for the history of coffee. That does not serve the study. | NTRODUCTION | 3 |
| Aspergillus niger was cultured on Potato Dextrose Agar (PDA) medium for 15 days at 25 2°C. Spores from pure culture suspended in sterilized distilled water containing 0.1% agar were used as an inoculum. The spore density was determined by using a hemacytometer to count 10 samples of fungal suspension. The calculated quantity of water required to raise the moisture content of the seed to 10% was supplemented with a spore suspension yielding a final concentration of approximately 2000-2500 spores/g. After 3, 6, and 9 months of storage at 0, 8, and 25 degrees Celsius, fatty acids were measured. | - the inoculum of fungus is not mentioned in the methodology. No microbiology at all in the manuscript. I would suggest to include the colony forming units (cfu) for fungus at various conditions to verify contamination. | MATERIALS AND METHODS | 3 |
| Done in the table. | - in the table, the legend isn't very clear. what is the units in the table ( it is not mentioned). if it is %, why they donot add up to 100%. | RESULTS | 7 |
| As the first reviewer suggested, this was explained, and the effect was emphasized. | - the conclusion should give us an idea of whether the quality has been preserved or not. | Conclusion | 12 |

**Response to the notes of the third reviewer:**

We thank the third reviewer for their dedicated work in providing various questions and recommendations. The table below lists these queries and ideas and our reactions to these changes and suggestions:

| After correction (Author’s response) | Before correction | Section | Page |
| --- | --- | --- | --- |
| Done | The method is not described appropriately. | MATERIALS AND METHODS | 4 |
| There are legends, which are written in bold to stand out. | Table legends are not present. | RESULTS |  |
| The statistical analysis was included in Tables S1-S8 in the supplementary materials, while Table 1 in the body of the manuscript contains a summary of these tables. | Tables need to include the results of the statistical analysis(i.e. p-value)  Statistical analyses data is not presented |  | supplementary materials |
| When conducting the current study, it was noted that there is a lack of research dealing with the effect of fungi on the type and content of fatty acids in coffee seeds. | Nothing in the discussion describe the effect of *A. niger* MH078571.1 | Discussion |  |
